# Reply to “The question of whether narcolepsy increases mortality should be answered based on reliable studies”

**DOI:** 10.1007/s44470-026-00121-8

**Published:** 2026-07-09

**Authors:** Amir Sharafkhaneh, Ahmed S. BaHammam, Yves Dauvilliers, Mehnaz Azarian, Michael Thorpy, Fang Han, Gulcin Benbir Senel, Murat Aksu, Javad Razjouyan

**Affiliations:** 1 Section of Pulmonary, Critical Care and Sleep Medicine, Department of Medicine, Baylor College of Medicine, Houston, TX USA; 2 Sleep Disorders and Research Center, Medical Care Line, Michael E. DeBakey VA Medical Center, Houston, TX USA; 3 Department of Medicine, College of Medicine, University Sleep Disorders Center, King Saud University, Riyadh, Saudi Arabia; 4 Sleep-Wake Disorders Unit, Gui-de-Chauliac Hospital, CHU Montpellier, Institute of Neurosciences of Montpellier, University of Montpellier, Montpellier, France; 5 Department of Medicine, Baylor College of Medicine, Houston, TX USA; 6 Center for Innovations in Quality, Effectiveness, and Safety, Michael E. DeBakey VA Medical Center, Houston, TX USA; 7 Albert Einstein College of Medicine, Bronx, NY USA; 8 Department of Sleep Medicine, Peking University People’s Hospital, Beijing, China; 9 Department of Neurology, Cerrahpasa Medical Faculty, Istanbul University-Cerrahpasa, Istanbul, Turkey; 10 Department of Neurology, Acibadem University, Istanbul, Turkey

**Keywords:** Retrospective study design, Narcolepsy, Mortality, Sleep apnea, Insomnia

We appreciate the letter by Mehri and Finsterer that outlines limitations often attributed to retrospective cohort designs. [1] Our manuscript explicitly acknowledges the inherent constraints of large-scale electronic health record (EHR) research, including residual confounding and the absence of cause-specific death data [2]. Several of the concerns enumerated by the authors, however, apply less directly to administrative EHR cohorts than to clinical chart reviews. Recall bias, for instance, is not a meaningful threat in datasets where exposures and outcomes are recorded prospectively at the point of care, independent of patient or investigator memory. Selection bias and confounding remain legitimate concerns, and we addressed both through propensity score matching on age, sex, race/ethnicity, and index diagnosis year, with adjustment for body mass index and Charlson Comorbidity Index. We agree with Talari and Goyal that retrospective designs cannot establish causality, and our manuscript explicitly frames the findings as associations rather than causal effects [3]. For rare conditions such as narcolepsy, EHR-based cohort studies remain indispensable because the prospective designs the authors propose are seldom feasible at the scale or duration needed to detect mortality signals. The Veterans Health Administration (VHA) vital status data have high accuracy and completeness (sensitivity 98.3% and exact-date agreement 97.6% relative to the National Death Index) [4]. These strengths support the reliability of our primary outcome assessment.

We agree with the authors that our narcolepsy diagnoses were not confirmed using polysomnography (PSG), MSLT, or CSF hypocretin measurements as required by ICSD-3 criteria for definitive NT1 diagnosis. The authors ask how many participants had cataplexy and whether all underwent PSG and CSF hypocretin testing. Because PSG and MSLT data are not captured in a standardized format across the national VHA dataset, and CSF hypocretin testing is rarely performed in routine VA practice, these data cannot be ascertained reliably from administrative records. This is precisely the constraint that motivated our computable phenotyping approach. We clearly stated that VHA PSG/MSLT data are not standardized nationwide and thus cannot be used for a consistent case definition; instead, we used a validated computable phenotyping algorithm requiring ≥ 2 ICD codes separated by 30–390 days to improve diagnostic specificity. This approach aligns with prior large EHR-based studies of narcolepsy and mitigates random miscoding [5]. We further note that even when PSG and MSLT are available, their reliability for distinguishing NT2 from idiopathic hypersomnia is limited; Trotti and colleagues reported that approximately half of patients with noncataplectic central hypersomnia received a different diagnosis on repeat MSLT [6]. The diagnostic gold standard is therefore imperfect even in tertiary referral settings. While individual NT1 features such as cataplexy or hypocretin deficiency cannot be confirmed in each patient, the purpose of our study was to evaluate real-world outcomes in clinically identified narcolepsy, not to revalidate diagnostic criteria.

We acknowledge that our analysis did not capture every potential comorbidity or medication class. The authors specifically raise endocrine and genetic disorders that may mimic hypersomnolence, along with antiepileptic drugs, analgesics, antihistamines, and immunosuppressants. We agree that endocrine conditions such as hypothyroidism can produce daytime sleepiness. However, these would more plausibly be misclassified within the general sleep clinic comparator than within the narcolepsy cohort, where the requirement of two narcolepsy ICD codes 30 to 390 days apart reduces the likelihood of such confusion. Concerning the medication classes named, none has a well-established direct effect on the mortality outcomes most likely operative in narcolepsy, namely cardiovascular events and accident-related deaths, and their inclusion would have introduced collinearity without addressing the principal causal hypotheses. Our study focused on comorbidity groups with established relevance to mortality risk in narcolepsy, namely cardiovascular, metabolic, pulmonary, neurologic, and psychiatric conditions. Expanding the scope to include every potential confounder would reduce interpretability, increase model instability, and exceed the constraints of available structured EHR data. Similarly, our medication analysis focused on pharmacological agents directly related to narcolepsy management.

We agree that a prospectively defined cohort design is essential for answering mechanistic questions and refining diagnostic precision. Such cohorts are logistically difficult to obtain for rare disorders and cannot provide the scale or 25-year follow-up available in the VHA population. In our study, we did not aim to establish causality but to assess whether narcolepsy, as diagnosed and managed in real-world clinical practice, is associated with increased mortality and healthcare utilization. The elevated mortality persisted even when the comparator general sleep clinic group had a substantially higher comorbidity burden at baseline, reinforcing the clinical relevance of our findings.

## Data Availability

Not applicable as this is a response to a letter submitted to JCSM.
